# Modeling Congenital Disorders of N-Linked Glycoprotein Glycosylation in *Drosophila melanogaster*

**DOI:** 10.3389/fgene.2018.00436

**Published:** 2018-10-02

**Authors:** Anna Frappaolo, Stefano Sechi, Tadahiro Kumagai, Angela Karimpour-Ghahnavieh, Michael Tiemeyer, Maria Grazia Giansanti

**Affiliations:** ^1^Istituto di Biologia e Patologia Molecolari del CNR, Dipartimento di Biologia e Biotecnologie, Sapienza - Università di Roma, Rome, Italy; ^2^Complex Carbohydrate Research Center, University of Georgia, Athens, GA, United States; ^3^Department of Biochemistry and Molecular Biology, University of Georgia, Athens, GA, United States

**Keywords:** Drosophila, glycosylation, congenital disorders, Golgi, model organism

## Abstract

Protein glycosylation, the enzymatic addition of N-linked or O-linked glycans to proteins, serves crucial functions in animal cells and requires the action of glycosyltransferases, glycosidases and nucleotide-sugar transporters, localized in the endoplasmic reticulum and Golgi apparatus. Congenital Disorders of Glycosylation (CDGs) comprise a family of multisystemic diseases caused by mutations in genes encoding proteins involved in glycosylation pathways. CDGs are classified into two large groups. Type I CDGs affect the synthesis of the dolichol-linked Glc_3_Man_9_GlcNac_2_ precursor of N-linked glycosylation or its transfer to acceptor proteins. Type II CDG (CDG-II) diseases impair either the trimming of the N-linked oligosaccharide, the addition of terminal glycans or the biosynthesis of O-linked oligosaccharides, which occur in the Golgi apparatus. So far, over 100 distinct forms of CDGs are known, with the majority of them characterized by neurological defects including mental retardation, seizures and hypotonia. Yet, it is unclear how defective glycosylation causes the pathology of CDGs. This issue can be only addressed by developing animal models of specific CDGs. *Drosophila melanogaster* is emerging as a highly suitable organism for analyzing glycan-dependent functions in the central nervous system (CNS) and the involvement of N-glycosylation in neuropathologies. In this review we illustrate recent work that highlights the genetic and neurobiologic advantages offered by *D. melanogaster* for dissecting glycosylation pathways and modeling CDG pathophysiology.

## Introduction

Protein glycosylation is one of the most frequent post-translational modifications in eukaryotes; approximately one fifth of all proteins in protein structural databases are glycosylated (Ohtsubo and Marth, 2006; Freeze and Ng, 2011; Khoury et al., 2011). The oligosaccharide moieties added to glycoproteins impact their structure and biological function by contributing to protein folding, stability, and transport to appropriate sub-cellular locations. Glycans also mediate cell–cell interactions, modulate signal transduction, and regulate molecular trafficking and endocytosis. The two main types of protein glycosylation are N-linked and O-linked glycosylation. The biosynthesis and elaboration of glycoprotein N-linked or O-linked glycans, require the coordinated action of hundreds of glycogenes, primarily glycosyltransferases and glycosidases, which are trafficked to specific locations within the endoplasmic reticulum (ER) and Golgi apparatus (Ohtsubo and Marth, 2006). Cytoplasmic and nuclear proteins are frequently modified with O-linked N-acetylglucosamine (GlcNAc) which regulates many biological processes but is beyond the scope of this review. N- and O-linked glycosylation of secreted and membrane protein starts in the ER or early *cis-*Golgi and is completed in later Golgi compartments. Major animal glycans contain ten monosacchararides: glucose (Glc), Galactose (Gal), N-acetylglucosamine (GlcNAc), N-acetylgalactosamine (GalNAc), fucose (Fuc), mannose (Man), xylose (Xyl), glucuronic acid (GlcA), iduronic acid (IdoA), and sialic acid (SA, either as 5-N-acetylneuraminic acid, Neu5Ac, or as 5-N-acetylglycolylneuraminic acid, Neu5Gc).

**FIGURE 1:**
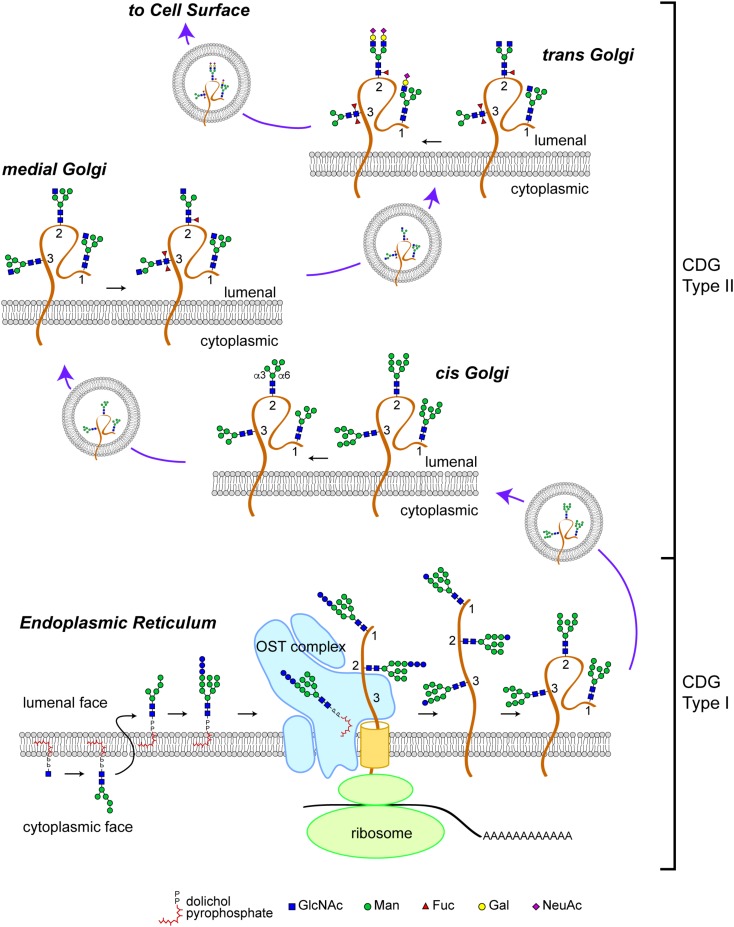
N-linked glycosylation pathway. Biosynthesis of the N-linked precursor glycan begins on the cytoplasmic face of the ER where a GlcNAc residue is added in a pyrophosphate linkage to dolichol, an isoprenoid lipid. The GlcNAc-P-P-Dol is extended to form Man_5_GlcNAc_2_-P-P-Dol which is then flipped so that the glycan moiety is within the lumen of the ER. Further extension produces a Glc_3_Man_9_GlcNAc_2_-P-P-Dol that is a substrate for the oligosaccharyltransferase (*OST*) complex, which transfers the precursor glycan *en bloc* to a nascent polypeptide. This figure depicts the glycosylation of a glycoprotein (brown) with 3 N-linked glycosylation sites (labeled *1, 2, and 3*). Once transfered to protein, the glycan precursor is trimmed of its Glc residues during folding as part of the calnexin/calreticulin quality control cycle. CDG Type I mutations affect the biosynthesis of the precursor glycan, its transfer to protein, and early trimming steps. Once successfully folded, glycoproteins bearing high-Man glycans are transported to the Golgi apparatus where Man trimming occurs. In the early *cis* Golgi, high-Man glycans can be trimmed to Man_5_GlcNAc_2_ by complete removal of Man residues on the α3 arm and partial removal of Man residues on the α6 arm. In the medial Golgi, the first committed step toward production of a complex glycan is taken; GlcNAcT1 adds a GlcNAc to the α3 Man residue to form a hybrid type glycan (*site 1* retains this structure). The GlcNAc-extended Man_5_GlcNAc_2_ glycan can be core fucosylated by the addition of a Fuc residue to the internal GlcNAc (*site 2*). In *Drosophila* and other arthropods, a second Fuc residue can be added (*site 3*). Additional Man trimming by Golgi mannosidases provide substrates for branching in the medial and *trans* Golgi (*site 2*). Subsequent extention with Gal and capping with sialic acid (shown here, as N-acetylneuraminic acid, NeuAc) completes the maturation of complex N-linked glycans. Hybrid glycans can also be extended on the α3 arm (*site 1*). The abundance of hybrid and complex glycans is reduced in *Drosophila* compared to vertebrate species due to the presence of an hexosaminidase that removes the GlcNAc added by GlcNAcT1, thereby blocking additional branching/extension and producing a paucimanose glycan (*site 3*). CDG Type II mutations impact the availability of substrates and the activity of enzymes that process N-glycans in the Golgi apparatus. Graphical representation of monosaccharide residues and glycan structures is consistent with the Symbol Nomenclature For Glycans (SNFG), which has been broadly adopted by the glycobiology community (Varki et al., 2015).

The N-glycosylation pathway starts at the ER membrane where a precursor glycan is built upon a dolichol isoprenoid lipid (**Figure 1**). This precursor glycan, with the composition Glc_3_Man_9_GlcNAc_2_, is transferred *en bloc* onto asparagine residues located within glycosylation sequons of nascent polypeptide chains either co-translationally or shortly after translation by multi-subunit oligosaccharyltransferase (OST) complexes within the lumen of the ER (Li et al., 2008; Zielinska et al., 2010; Shwartz and Aebi, 2011). The transferred Glc_3_Man_9_GlcNAc_2_ glycan is subsequently trimmed by sequential action of ER glucosidases. Glc-trimming is an essential component of the folding process for most secretory pathway glycoproteins (Helenius and Aebi, 2004). Thus, human diseases that arise from altered biosynthesis or trimming of the N-linked precursor glycan, or ineffective transfer of the precursor to protein will impact the folding and stability of many glycoproteins and, consequently, manifest with multi-systemic and broadly severe clinical phenotypes.

Glycoproteins arrive at the *cis-*Golgi carrying high-Man glycans (**Figure 1**). Mannose trimming in the *cis-*Golgi by Golgi α-mannosidases removes Man residues to generate the Man_5_GlcNAc_2_ intermediate. In medial Golgi compartments, Man_5_GlcNAc_2_ is the substrate for GlcNAcT-1, a glycosyltransferase that transfers a GlcNAc residue to a terminal Man residue on the α3-arm of the Man_5_GlcNAc_2_ structure, thereby initiating the synthesis of hybrid and complex N-linked glycans (Stanley, 2011; Moremen et al., 2012). The product of GlcNAcT-1 is also a substrate for core fucosylation, the addition of one or more Fuc residues (depending on the species) to the most proximal core GlcNAc residues attached to Asn. The α3-arm initiated by GlcNAT-1 can be extended with Gal, Sia and/or other residues, resulting in the production of hybrid structures. Removal of the remaining Man residues from the α6-arm allows branching with additional GlcNAc residues catalyzed by specific GlcNAcT enzymes and subsequent extension to generate fully elaborated multiantennary complex glycans in late medial and *trans* Golgi compartments (Stanley, 2011). The vectorial nature of N-glycan processing is facilitated by enzyme specificity and by the spatial distribution of processing steps across the Golgi apparatus. Therefore, human diseases that impact N-glycan fine structure may arise from genes that encode for processing enzymes or for proteins that regulate Golgi architecture and trafficking. Such diseases may be characterized by relatively restricted phenotypes associated with altered function, half-life, or targeting of specific glycoproteins.

In contrast to N-linked glycosylation, O-linked glycosylation does not rely on a precursor core that is transferred *en bloc* to the nascent polypeptide. Instead, O-glycosylation is initiated on folding or folded proteins and involves the formation of a glycosidic linkage between serine or threonine and GalNAc, GlcNAc, Man, Glc, Xyl, or Fuc residues (Stanley, 2011). Some *O*-glycans are specifically elaborated on well-defined protein domains and contribute to protein folding, stability, protease sensitivity, and protein function. The biosynthesis of *O*-glycans in the secretory pathway is initiated in the early *cis-*Golgi or in a transitional compartment that retains characteristics of the ER and is completed in subsequent processing steps distributed across the Golgi apparatus. Therefore, human diseases arising from genes that regulate Golgi dynamics may impact both N-linked and O-linked glycoprotein glycosylation.

Congenital disorders of glycosylation (CDGs) are inborn errors in protein and lipid glycosylation that arise from mutations in genes controlling steps in glycan addition (Freeze and Ng, 2011; Jaeken, 2013). More than 100 distinct forms of CDGs have been discovered, many of which display multisystemic defects including severe neurological impairment, highlighting the important role of regulated glycosylation in central nervous system (CNS) functions (Barone et al., 2014; Freeze et al., 2015). Because 1–2% of the genome encodes for glyco-enzymes and glycan transporters, it is likely that many other CDGs remain to be discovered (Freeze et al., 2015). CDGs have been traditionally divided into two large groups (Goreta et al., 2012; Freeze et al., 2014). Type I CDGs impair the synthesis of the dolichol pyrophosphate oligosaccharide precursor of N-linked glycoproteins or its transfer to acceptor proteins (Freeze and Ng, 2011) resulting in decreased efficiency of protein N-glycosylation. Type II CDGs (CDG-II) are characterized by defects in the processing of N-linked glycans or the biosynthesis of O-linked oligosaccharides (Freeze and Ng, 2011; Goreta et al., 2012; Freeze et al., 2014). Although most CDGs exhibit neurological impairment (Freeze et al., 2015), there are no comprehensive studies, aimed at elucidating the molecular mechanisms that link defective glycosylation to the neuropathological aspects of the disease. Animal models that faithfully recapitulate the pathological aspects of the disease, including the neurological defects, provide a valuable resource to study the molecular mechanisms underlying pathology in CDGs. In this review we focus our attention on the advantages offered by the use of *Drosophila melanogaster* for understanding and modeling the glycobiology of CDGs.

## *Drosophila melanogaster* as a Model System for Studying Cdgs

*Drosophila* models offer many advantages for studying CDGs as well as other human diseases (Moulton and Letsou, 2016). Fundamental biological processes are highly conserved between *Drosophila* and humans; approximately 75% of human-disease related genes have a homolog in *Drosophila* (Reiter et al., 2001; Chien et al., 2002). Moreover the genome of *D. melanogaster* is far less complex than the human genome and exhibits fewer gene duplications (Hartl, 2000). All of these characteristics, and its extraordinary repertoire of readily available genetic tools, have combined to make *Drosophila* a valuable, emerging model system for investigating glycan-dependent functions *in vivo* and for understanding the link between CDG neuropathology and glycan changes (Katoh and Tiemeyer, 2013; Scott and Panin, 2014). Such studies are extremely challenging in vertebrates due to the complexity of the nervous system and the redundancy of glycosylation pathways and enzymes (Freeze et al., 2014; Scott and Panin, 2014). *Drosophila* combines the advantages of a well-characterized glycome and the availability of electrophysiological and behavioral assays to test neurological impairment in the whole organism (Gatto and Broadie, 2011; Dani et al., 2014; Scott and Panin, 2014). Moreover, larval neuromuscular junction (NMJ) synapses use ionotropic glutamate receptors (GluRs), providing an excellent model system for excitatory synapses in the mammalian CNS.

**FIGURE 2:**
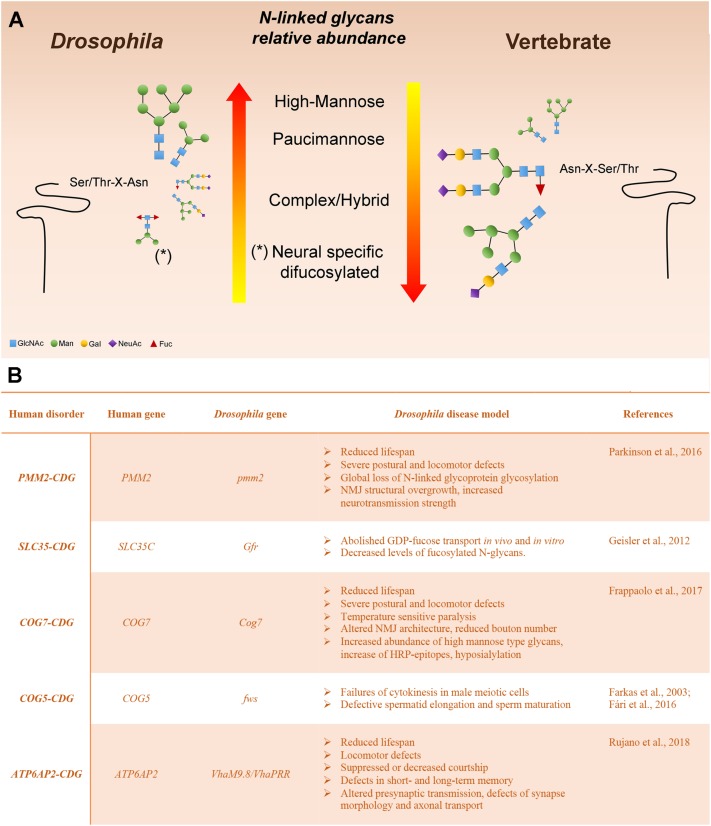
*Drosophila melanogaster* as a model system to study glycoprotein N-glycosylation **(A)** Representation of *Drosophila* and vertebrate N-glycome characteristics. N-linked glycans are scaled proportionally to their relative abundance. The major reason for the high-mannose and pauci-mannose dominance in the *Drosophila* N-linked glycan profile is the existence of an arthropod-specific, N-acetylhexosaminidase known as Fused lobes (Fdl), which converts the precursor for complex glycans (GlcNAc_1_Man_3−5_GlcNAc_2_-Protein) to a paucimannose structure (Man_3−5_GlcNAc_2_-Protein) that cannot be extended further. Glycoprotein glycans that escape Fdl are fully capable of being processed into complex structures. **(B)** Human glycosylation disorders and phenotypic characteristics of the *Drosophila* model.

Various analytic techniques have revealed that the most abundant N-linked glycans on *Drosophila* glycoproteins are of the high-Man or paucimannosidic type. However, hybrid and complex glycans are also present, although they represent a lower fraction of the total glycan profile when compared with vertebrates (Katoh and Tiemeyer, 2013). The relative paucity of complex glycans in *Drosophila* is a result of an arthropod-specific glycan processing enzyme encoded by a gene named Fused Lobes (*Fdl*) that removes the GlcNAc residue added by GlcNAcT-1, thereby blocking further glycan elaboration (**Figures 1**, **2**). The presence of Fdl in the secretory pathway means that *Drosophila* glycan profiles are skewed away from the highly abundant complex profiles found in most vertebrates. Nonetheless, the glycans that escape the activity of Fdl in *Drosophila* are readily processed to complexity, indicating common logic underlies glycan maturation in vertebrates and *Drosophila.* The resulting low content of complex glycans also provides a benefit for this system because it generates a simpler profile to analyze and a larger dynamic range for detecting shifts induced by mutations. Additionally, unlike mammalian organisms, the *Drosophila* genome contains only a single sialyltransferase (*DSiaT*), which greatly simplifies *in vivo* analysis of glycoprotein sialylation (Aoki et al., 2007; Koles et al., 2007; Repnikova et al., 2010).

In this section we describe *Drosophila* mutants that offer functional models for characterized human CDGs (**Figure 2**). We discuss the phenotypic characteristics that recapitulate the pathological aspects of the human disease and the translational impact of modeling the CDG in this organism. Many other *Drosophila* mutants have been shown to impact glycoprotein or glycolipid glycosylation and it is likely that the impact of many others is underappreciated (Seppo et al., 2003; Baas et al., 2011; Daenzer et al., 2016; Jumbo-Lucioni et al., 2016). But here, we focus on those mutations that have immediate parallels with human type I and II CDGs.

### PMM2-CDG (CDG-Ia)

The most prevalent CDG is known as CDG-1a or PMM2-CDG, accounting for around 80% of all diagnosed cases. PMM2-CDG is inherited as an autosomal-recessive trait resulting from mutations in human *PMM2*, which encodes phosphomannomutase-2, that converts mannose-6-phosphate to mannose-1-phosphate, the precursor of GDP-mannose (Freeze et al., 2014, 2015). Because GDP-mannose is the donor for the addition of the first 5 Man residues to the dolichol-linked precursor, synthesis of N-linked glycans is impacted, as is *O*-mannosylation. The defect results in hypoglycosylation of many types of glycoproteins including serum glycoproteins, plasma membrane glycoproteins, and lysosomal enzymes. Pediatric patients suffering with CDG-Ia present with variable clinical features that affect nearly all systems and include failure to thrive, hypotonia, psychomotor retardation, ataxia, dysmorphia and coagulopathy (Freeze, 2009; Grünewald, 2009; Jaeken, 2013). Most adult CDG-Ia patients are wheelchair bound and display peripheral neuropathy and mental retardation (Grünewald, 2009).

Overall, the *Drosophila* PMM2 protein displays 56% amino acid identity with human PMM2 (Parkinson et al., 2016). Parkinson et al. (2016) generated *Drosophila pmm2* mutants. Similar to CDG-1a patients, *pmm2* mutants displayed uncoordinated movement and reduced lifespan. Analysis of the N-linked glycome of the *pmm2*-null mutant larval body demonstrated a global suppression of N-linked glycosylation. Furthermore, the N-linked glycome of adult heads with neurally targeted *pmm2* RNAi revealed increased abundance of pauci-mannose glycans. Analysis of the larval NMJs revealed altered glycan composition within the heavily glycosylated synaptomatrix which correlated with striking NMJ structural overgrowth and increased neurotransmission strength. Since NMJ synaptogenesis requires *trans*-synaptic Wnt/Wingless signaling, which in turn depends on expression of Dally-like protein (Dlp, a heparan sulfate proteoglycan) and matrix metalloproteinases (MMPs). Because knockdown of *PMM2* resulted in loss of MMP2, reduced synaptic levels of Wingless, Dlp co-receptor and downstream *trans-*synaptic signaling, the authors propose that the matrix metalloproteome and Wnt signaling pathway might provide potential new targets for developing CDG-1a treatments.

### SLC35-CDG (CDG-IIc)

Also known as CDG-IIc and leukocyte adhesion deficiency type II syndrome (LAD II), this disorder is caused by mutations in a GDP-Fuc transporter (GFR). SLC35-CDG patients show craniofacial dysmorphism, severe retardation and chronic infections with unusually high leukocytosis. Importantly neutrophils of these patients lack the ability to synthesize the fucosylated glycan sialyl-Lewis X, a ligand of the selectin family of cell adhesion molecules, that is necessary for their recruitment to infection sites (Yakubenia et al., 2008). Several mutations in SLC35 nucleotide sugar transporters have been identified in *Drosophila*, including the ER Fuc transporter (*Efr*), fringe connection (*frc*), slalom (*sll*) and neurally altered carbohydrate (*nac*). In *nac*^1^ mutant, a conserved serine at position 29 of the Golgi GFR is replaced by a leucine, which abolishes GDP-Fuc transport *in vivo* and *in vitro*. Mass spectrometry and HPLC analysis demonstrated reduced core α1,3-and α1,6-fucosylation in *nac*^1^ (Geisler et al., 2012). While Lewis-type glycans have not yet been identified in *Drosophila*, the commonality of altered fucosylation in SLC35 CDG-IIc and in select *Drosophila* mutants provides opportunities to investigate the regulation of protein fucosylation in a whole organism.

### COG-CDG (CDG-IIe and CDG-IIi)

The eight-subunit Conserved Oligomeric Golgi (COG) complex is a Golgi tether required for intra Golgi trafficking of vesicles that recycle Golgi resident proteins and is essential for proper localization of Golgi-localized glycosylation enzymes including glycosyltransferases (Ungar et al., 2002; Kranz et al., 2007; Miller and Ungar, 2012; Willett et al., 2013; Climer et al., 2015). Mutations in the genes encoding human COG1, COG2, and COG4–COG8 are associated with monogenic forms of inherited, autosomal recessive, CDGs-II (Freeze and Ng, 2011; Climer et al., 2015). Common features of patients carrying mutations in COG proteins (COG-CDG) are feeding problems and developmental defects, including microcephaly and growth retardation associated with dysmorphic features, hypotonia and cerebral atrophy (Wu et al., 2004; Spaapen et al., 2005; Foulquier et al., 2006; Kranz et al., 2007; Morava et al., 2007; Ng et al., 2007; Paesold-Burda et al., 2009; Reynders et al., 2009; Zeevaert et al., 2009; Lübbehusen et al., 2010; Fung et al., 2012; Kodera et al., 2015). COG7–CDG patients had the highest mortality within the first year of life and presented with dysmorphic facial features, generalized hypotonia, skeletal anomalies, hepatomegaly, progressive jaundice, cardiac insufficiency, microcephaly, and severe epilepsy (Wu et al., 2004; Spaapen et al., 2005; Morava et al., 2007; Ng et al., 2007; Zeevaert et al., 2009). Defects in COG proteins have been linked to glycosylation alterations in mammalian cultured cells and in COG–CDG patients, including hyposialylation of serum proteins, abnormal synthesis of N- and O-linked glycans and altered glycolipid glycosylation (Kingsley et al., 1986; Suvorova et al., 2002; Wu et al., 2004; Spaapen et al., 2005; Morava et al., 2007; Ng et al., 2007; Zeevaert et al., 2009; Struwe and Reinhold, 2012).

Analysis of phenotypes associated with mutations in *Drosophila* homologs of human COG complex members highlights both the value and also the limitations of modeling COG-complex disorders in this organism. The *Drosophila* homolog of human COG5, Four way stop (Fws), is not essential for adult survival but is required for male fertility. Mutations in *fws* impair spermatocyte cytokinesis, acroblast structure and elongation and individualization of differentiating spermatids (Farkas et al., 2003; Fári et al., 2016). Thus, the *Drosophila COG5* mutant presents less severe involvement than the presently known human COG-complex CDGs. On the other hand, loss of COG7 in COG7–CDG patients and in *Drosophila* mutants results in reduced life span and severe psychomotor defects (Frappaolo et al., 2017). Analysis of N-glycans from heads of *Drosophila Cog7* mutants, revealed increased abundance of high-Man type glycans compared to wild type, accompanied by a disproportionate increase of the Man_5_GlcNAc_2_ glycan, which is the precursor for all complex glycans. Additionally, a substantial increase in the abundance of a family of neural-specific, difucosylated N-glycans known as HRP-epitopes, was detected. However, not all N-glycans were increased in *Cog7* mutants. A single sialylated N-glycan was detected among the glycans harvested from adult heads and quantification relative to standard indicated that it was decreased in two mutant allelic combinations compared to wild type (Frappaolo et al., 2017). Moreover, like *DSiaT* mutants, *Cog7* mutant flies exhibit temperature sensitive (TS) paralysis, coordination defects, and altered architecture of larval NMJ. Thus the phenotypic characteristics of our *Drosophila* COG7–CDG model closely parallel the pathological characteristics of COG7–CDG patients including N-linked glycome defects with hyposialylation. Ongoing analysis of the COG protein interactome is beginning to highlight molecular hierarchies and trafficking paradigms that may underlie altered protein glycosylation (Belloni et al., 2012; Miller et al., 2012; Willett et al., 2013; Hong and Lev, 2014; Climer et al., 2015; Bailey Blackburn et al., 2016; Frappaolo et al., 2017; Sechi et al., 2017).

### ATP6AP2-CDG

The multi-subunit vacuolar-type proton ATPase (V-ATP-ase) is a highly conserved proton pump, which acidifies intracellular compartments and is essential for endocytosis and vesicular trafficking. Rujano et al. (2018) identified missense mutations in the extracellular domain of the accessory V-ATPase subunit ATP6AP2 that cause a novel glycosylation disorder associated with hepatopathy, immunodeficiency, cutis laxa, muscular hypotonia, dysmorphic features, and psychomotor impairment. Analysis of ATP6AP2-CDG patients’ serum proteins revealed hypoglycosylation, a defect that could be recapitulated by *ATP6AP2* deficiency in the mouse. Null alleles of the *Drosophila* ortholog of *ATP6AP2* cause an early lethal phenotype in *Drosophila*. The introduction of an ATP6AP2 transgene carrying the p.L98S mutation in the background of *Drosophila ATP6AP2* null mutants, reduced viability and affected the developing optic lobes in larval brains by expanding the pool of optic lobe neuroblasts, a phenotype associated with altered Notch signaling (Vaccari et al., 2010). In agreement with the role of V-ATPase-mediated acidification in autophagic degradation (Mauvezin et al., 2015), p.L98S mutation leads to lipid accumulation and autophagic dysregulation in the liver-like fat body, associated with defects of lysosomal acidification and mTOR signaling. Thus the *Drosophila* ATP6AP2-CDG model has allowed the elucidation of molecular mechanisms underlying pathological aspects of the human disease.

## Conclusion and Perspectives

Many impactful studies utilizing model systems (Zebrafish, flies, *C. elegans*, mice, etc.,) have enhanced our understanding of the underlying biochemical and phenotypic consequences of altered glycan biosynthesis associated with human CDG subtypes. Clinical phenotypes of human CDGs have parallels in these model systems. For the growing subset of CDGs modeled in *Drosophila*, specific phenotypes related to neural function, lifespan, viability, and glycomic diversity are replicated across these highly divergent species. This phenotypic reproducibility across species should not be surprising since the core biosynthetic pathways for protein glycosylation as well as the basic mechanisms that regulate Golgi trafficking are shared across broad swaths of evolutionary space. This conservation will allow mechanistic questions to be effectively answered in CDG models. One of these key questions is whether phenotypes arise from altered glycosylation of broad sets of glycoproteins or whether aberrant glycosylation of small subsets of glycoproteins can be linked to underlying pathologies. Once candidate proteins, whose glycosylation is altered in a given CDG, are identified by cutting edge glycoproteomics, the genetic tools offered by model systems such as *Drosophila* will allow unprecedented targeted investigations of the cell- and tissue-specific impacts of glycosylation deficiencies.

## Author Contributions

All authors edited and critically revised the manuscript.

## Conflict of Interest Statement

The authors declare that the research was conducted in the absence of any commercial or financial relationships that could be construed as a potential conflict of interest. The handling Editor and author MG declared their involvement as co-editors in the Research Topic, and confirm the absence of any other collaboration.
